# Low‑to‑moderate hyperoxia in animal models: is there evidence for harm?

**DOI:** 10.1186/s40635-023-00540-4

**Published:** 2023-08-28

**Authors:** Thijs A. Lilien, Reinout A. Bem

**Affiliations:** Department of Pediatric Intensive Care, Emma Children’s Hospital, Amsterdam UMC Location University of Amsterdam, Amsterdam, The Netherlands

**Dear editor,**

With great interest we have read the meta-analysis of *Minkove *et al*.* on the potential effects of low-to-moderate hyperoxia on lung injury in animal models [1]. They address potential harm from moderate hyperoxia (FiO_2_ ≤ 0.6) exposure in the critical care setting, an important topic which has received limited attention as compared to exposure to severe hyperoxia (FiO_2_ > 0.6). With the current general protective strategy to avoid severe hyperoxia and the resulting extremes of hyperoxemia, it remains highly uncertain whether moderate hyperoxia contributes to clinically relevant harm [2]. As preclinical research may inform clinical practice, the authors should be commended for their extensive search and review. However, we believe a bit more nuance in their report would have been appropriate.

First, it was implied that the data suggest that moderate hyperoxia may be harmful, but the presented data with statistical analysis do not clearly support this conclusion. After all, the pooled effect estimates of the primary analysis on survival and secondary analysis on lung weight do not provide significant results. Second, we feel that the authors did not sufficiently address the tendency of random-effects models to overestimate the pooled effect estimate, especially if the number of studies is small [3]. In an attempt to account for this limitation, we have tried to reproduce the previous analyses and added a Knapp–Hartung adjustment of the pooled confidence interval (CI) [4]. In both analyses this adds a degree of uncertainty to the pooled effect estimate (Table 1), further challenging the suggestion that moderate hyperoxia may be harmful. The report also did not include a CI of the *I*^*2*^-statistic and re-analysis reveals that in both analyses the level of heterogeneity is still highly uncertain (Table 1). Since heterogeneity estimates are often difficult to interpret, it has been recommended to calculate the prediction interval to estimate the range wherein the effect estimate of a new study would fall [5]. Accordingly, it can be observed that new studies are likely to produce inconclusive results regarding the association of moderate hyperoxia with outcome (Table 1). Publication bias resulting from inconclusive or negative results in particular from animal studies is generally an important limitation. Yet, it was suggested that new experimental studies are warranted to examine the effects of moderate hyperoxia. We, however, respectfully argue against such additional animal studies. Sample sizes varying from 300 to 900 animals would be required to have 80% power to reject the null hypothesis that moderate hyperoxia exposure is not associated with survival. For a complex, clinically relevant model, it would probably be necessary to study larger animals. Following the three R principle (replacement, reduction and refinement) in animal research, we feel that the data and calculated prediction interval would not automatically support such experiments with most likely inconclusive results. Previous pragmatic trials on oxygen therapy assessing survival in critically ill adults have also so far yielded inconclusive results [2]. Instead of investigating survival further, perhaps it would be better to focus on whether (moderate) hyperoxia is associated with subtler, long-term effects in a ‘bedside-to-bench’ approach, a topic still relatively little studied beyond premature infants.

**Table 1:** Re-analysis of pooled effect sizes including Knapp–Hartung adjustment and prediction interval

	Original paper^a^ (95% CI)	Including K–H adj^b^ (95% CI)	*I*^2^-statistic (95% CI)	Prediction interval (95% CI)
Survival, OR	0.37–1.25	0.33–1.39	0–75%	0.29–1.61
Lung weight, SMD	− 0.06–1.00	− 0.16–1.19	0–79%	− 0.33–1.36

^a^Minkove et al. 2023; ^b^Knapp–Hartung adjustment of the pooled confidence interval. *CI* confidence interval, *OR* odds ratio, *SMD* standardized mean difference

## Data Availability

The supporting data in this letter were extracted from *Minkove *et al*.* (2023) Intensive Care Med Exp. Supporting data are available upon request.
